# Effects of nitro-butoxyl- and butyl-esters of non-steroidal anti-inflammatory drugs compared with parent compounds on the contractility of digital arterial smooth muscle from the fallow deer (Dama dama)

**DOI:** 10.1007/s10787-021-00858-z

**Published:** 2021-09-16

**Authors:** Brian A. Callingham, M. Akram Khan, Anthony S. Milton, K. D. Rainsford

**Affiliations:** 1 Department of Pharmacology, University of Cambridge, Tennis Court Road, Cambridge, CB2 1PD UK; 2 Biomedical Research Centre, Sheffield Hallam University, Howard Street, Sheffield, S1 1WB UK

**Keywords:** NSAIDs, Nitric oxide, Arterial, Deer, Smooth muscle, Gastrointestinal, Cardiovascular, NO-NSAIDs

## Abstract

**Background:**

Non-steroidal anti-inflammatory drugs (NSAIDs) are a major cause of upper gastro-intestinal (GI) ulceration and bleeding as well as cardiovascular (CV) diseases (e.g., myocardial infarction and stroke). A feature common to both these adverse events is a variety of vascular reactions. One approach to overcome these side effects has been the development of nitric-oxide (NO)-donating NSAIDs. The NO is considered to overcome some of these vascular reactions caused by NSAIDs. Unfortunately, the NO-NSAIDs developed so far have not had the expected benefits compared with NSAIDs alone.

**Objectives:**

Using in vitro preparations it is hoped to gain insight into the vascular and smooth muscle reactions induced by NO-NSAIDs compared with NSAIDs as a basis for improving the protective responses attributed to the NO-donating properties of these drugs.

**Methods:**

A range of NO-NSAIDs was synthesized based on the esterification of NSAIDs with the nitro-butoxylate as a prototype of an NO-donor. These compounds, as well as NO-donor agents and NSAIDS, were examined for their possible effects on isolated segments of digital arteries of fallow deer, which provide a robust model for determining the effects of vasodilator and vasoconstrictor activities, in comparison with those of standard pharmacological agents.

**Results:**

The NO-NSAIDs were found to antagonise the smooth muscle contractions produced by 5-hydroxytryptamine (serotonin, 5-HT). However, while almost all their parent NSAIDs had little or no effect, with the exception of the R-(−)-isomers of both ibuprofen and flurbiprofen, which caused vasodilatation, all the NO-NSAIDs tested antagonised the increase in tension produced by 5-HT.

**Conclusions:**

R-(−)-ibuprofen and R-(−)-flurbiprofen, along with the nitro-butoxyl esters of the NSAIDs examined, produce relaxation of segments of deer digital artery smooth muscle in vitro*.* The evidence presented suggests that their mechanism involves the release of NO or its products.

## Introduction

Non-steroidal anti-inflammatory drugs (NSAIDs) are amongst the most widely used drugs for prescription and non-prescription (‘over-the-counter’ or OTC) medications for the treatment of musculo-skeletal and various acute and chronic painful and inflammatory conditions (Rainsford 2007). Their use is associated with the development of serious adverse drug reactions (ADRs) especially in the gastro-intestinal (GI) tract of elderly patients with compromised health status (Rainsford et al. 2008; Lanas 2010; Lanas et al. 2010; Rahme and Bernatsky 2010) or those with compromised cytochrome CYP2C9 metabolism (Carbonell et al. 2010); Süleyman et al. 2007). Over recent decades there has been increasing concern about the risks of NSAIDs, especially the cyclo-oxygenase (COX)-selective agents or coxibs, being associated with cardiovascular (CV) and cerebrovascular reactions including increased risk of myocardial infarction (Antman et al. 2007; McGettigan and Henry 2011; Olsen et al. 2012; Shau et al. 2012; Sudano et al. 2012; Caughey et al. 2011) and stroke (Barthélémy et al. 2011; Caughey et al. 2011; Varas-Lorenzo et al. 2011). These reactions are primarily related to hypertension that is exacerbated by NSAIDs (Barthélémy et al. 2011; Varas-Lorenzo et al. 2011) as well as T-cell associated plaque-instability in atherosclerosis (Padol and Hunt 2010; Rainsford 2010). The atherogenic promoting effects of NSAIDs may also be related to their propensity to divert arachidonic acid through the 5-lipoxygenase pathway (Yu et al. 2012).

Current concerns regarding the safe use of NSAIDs have centred on the combined GI and CV risks of these drugs (Lanas et al. 2010; Scheiman and Hindley 2010; Salvo et al. 2011). A general feature that is common to both these adverse reactions is the effects of the NSAIDs on vascular reactions. Thus, in addition to the abovementioned vascular effects in CV disease, NSAIDs also cause microvascular injury in the early stages of the development of gastric mucosal damage (Rainsford 1983, 1992, 1993a, b; 1999; Gyömber et al. 1996a, b; Pasa et al. 2009; Tarnawski et al. 2012). The NSAID-induced impairment of platelet aggregation contributes to the extravasation of blood from the damaged microvasculature into the interstitial space, ischaemia and subsequent bleeding that accompanies the pathological injury to the gastric mucosa (Rainsford 1986, 1992; Gyömber et al. 1996a, b; Tarnawski et al. 2012). The initiation of vascular constriction by NSAIDs is considered to be related to excess production of vasoconstrictor peptido-leukotrienes which occurs from the diversion of arachidonic acid through the 5-lipoxygenase pathway as a result of NSAIDs inhibiting the cyclo-oxygenases (Rainsford 1986, 1993a, b, 1999; Rainsford et al. 1995; Gyömber et al. 1996a, b). This is accompanied by accumulation, endothelial interactions and activation of polymorphonuclear (neutrophil) and other leucocytes that contribute to mucosal damage (Rainsford et al. 1995; 2012; McCafferty et al. 1995; Appleyard et al. 1996; Wallace and Cirino 1994; Wallace 1997; Wallace et al. 1999; Muscará, et al. 2000). Aside from arachidonate metabolites (prostanoids, leukotrienes, lipoxins), nitric oxide (NO) is known to have a central role in the control of vascular smooth muscle contraction, blood flow and platelet-endothelial interactions (Brzozowski et al. 2008; Palileo and Kaunitz 2011; Tarnawski et al. 2012). However, it has also been shown that NO has actions that could be seen to be protective in the GI tract, by reducing vascular injury, enhancing production of protective mucus, reducing the effects of acid-pepsin and promotion of anti-thrombotic effects (Wallace et al. 1993, 1994, 1999; Fiorucci and Distrutti 2011).

To this end, a range of NSAIDs coupled to an NO-releasing moiety (NO-NSAIDs) has been developed, in the hope that such compounds, by releasing NO in the mucosa, would be less damaging to the GI tract. Some of these NO-NSAIDs have been shown experimentally to cause less gastric injury than the parent NSAIDs (Wallace et al. 1994, 1999; Fiorucci and Distrutti 2011; Gund et al. 2014). The actions of these drugs in preventing GI injury are considered to result from the hydrolysis of an NO-ester link. Despite an immense amount of research, the outcomes from the long-term studies with candidate NO-NSAIDs (e.g. NO-naproxen or naproxcinod) have been disappointing (Milton et al. 1999; Lowry 2010), although NO-aspirin may have potential as an anti-thrombotic agent (Wallace et al. 1999; Callingham et al. 2012). In the present study, the actions of some NO-NSAIDs were compared with established NSAIDs and NO-donating analogues on the contractility of isolated segments common digital arteries of fallow deer (Dama dama; Callingham et al. 2012).

## Methods

Unless otherwise stated, NSAIDs, together with the intermediates used in the synthesis of the nitrobutoxy compounds described below, were obtained from Sigma-Aldrich (Poole, Dorset, UK). 5-Hydroxytryptamine (serotonin, 5-HT), phenylephrine (PHE), the soluble guanylate cyclase (sGC) inhibitor 1H-[1,2,4]Oxadiazolo[4,3-a]quinoxalin-1-one (ODQ) and other laboratory reagents were also obtained from Sigma-Aldrich (Poole, Dorset, UK).

The propionic acids, ibuprofen and flurbiprofen, are referred to as their racemic mixtures (*rac*). The R-(−)- and S-( +)-isomers of these drugs were gifts from Boots Healthcare International, Nottingham, UK.

### Chemistry

**Fig. 1:**
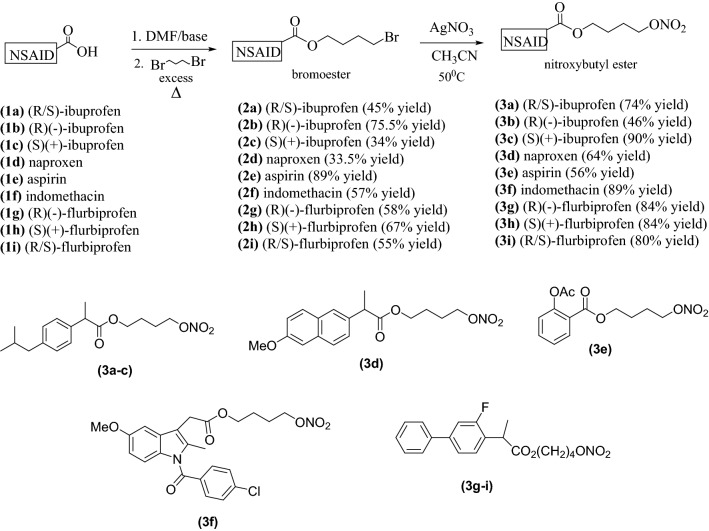
General scheme for the synthesis of NO-NSAIDs **(3a**–**i)** in two steps by S_N_2 reactions

**Fig. 2:**
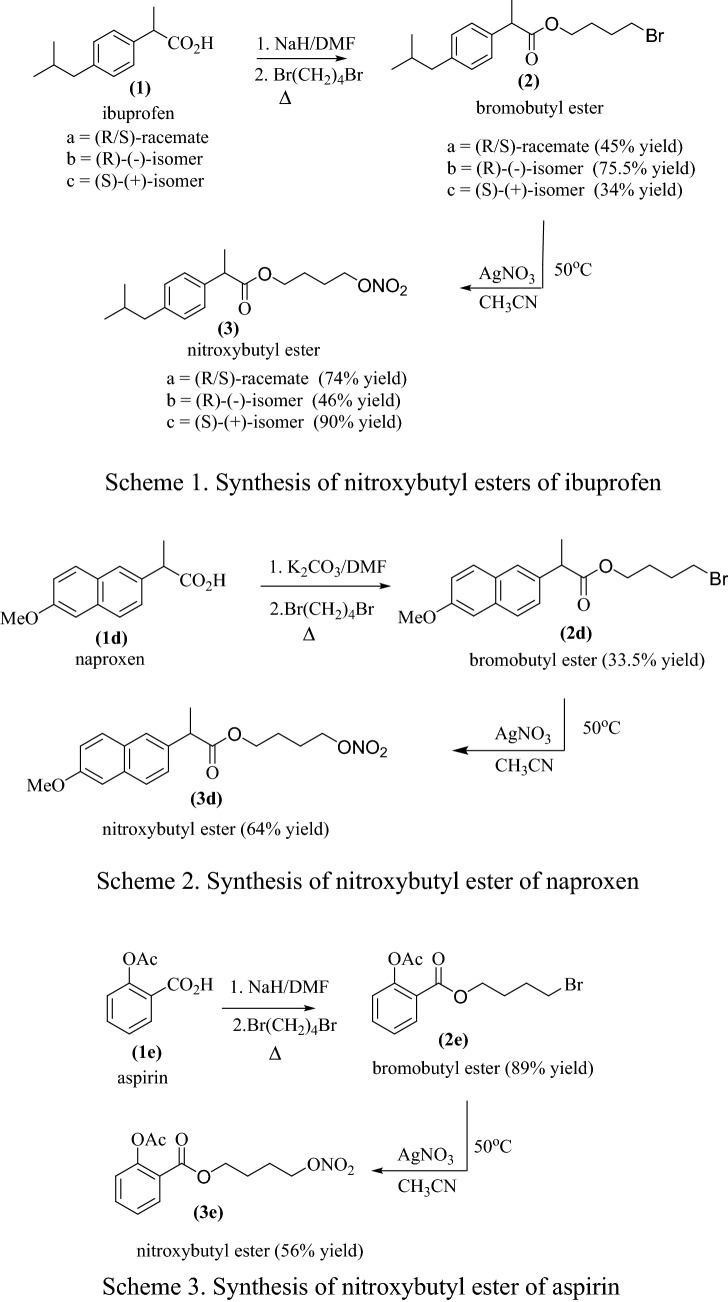

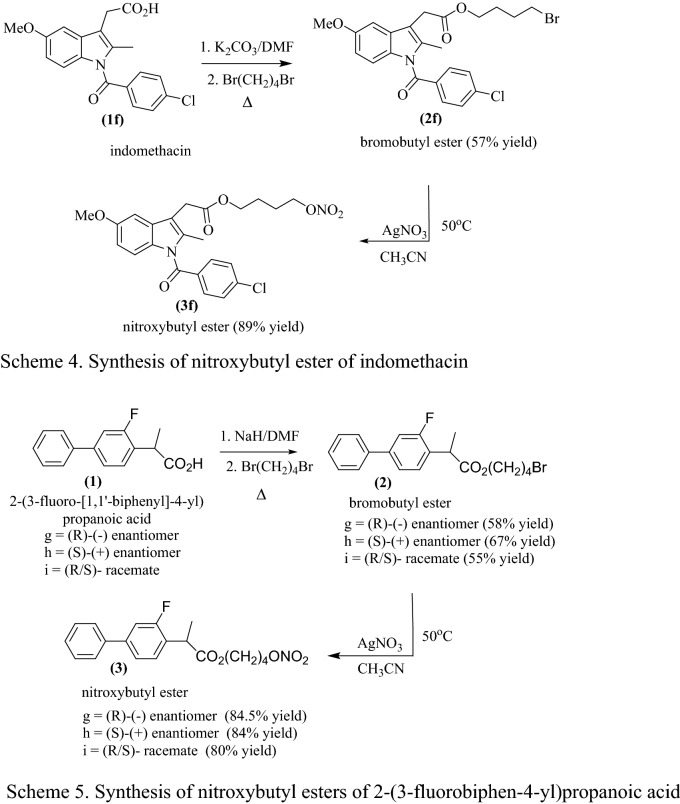
Summary of the synthesis of NO-NSAIDs by a method modified from that of Wallace et al (1994, 1995). a: R/S-ibuprofen, b: R-(-)-ibuprofen, c: S-(+)-ibuprofen, d: S-(+)-naproxen, e: aspirin (Schemes 1-3), f: indomethacin (Scheme 4), g: R-(-)-flurbiprofen), S-(+)-flurbiprofen and R/S-flurbiprofen (Scheme 5)

The NO-NSAIDs **(3a**–**i),** were synthesized by a modification of the literature method (Wallace and Cirino 1994; Wallace et al. 1995) that is shown in Figs. 1 and 2.

#### General methods

Melting points are uncorrected and were determined on Stuart Scientific SMP3 apparatus. Infrared spectra were recorded with an ATI Mattson Genesis series FTIR spectrophotometer. ^1^H NMR and ^13^C NMR spectra were recorded in CDCl_3_ using a Brucker AC 250 spectrometer operating at 250 and 62.9 MHz, respectively. Chemical shifts (δ) are recorded in ppm downfield from Me_4_Si as internal standard and J values are given in Hz. Mass spectra were recorded with EI-VG 7070E mass spectrometer. Accurate masses were determined on VG Autospec EI mass spectrometer with magnetic sector instrument. Optical rotations were measured at 23 °C with a Bellingham and Stanley ADP 440 polarimeter using dichloromethane as the solvent. All solvents were dried and distilled by standard techniques.

*Typical procedure for the preparation of the bromobutyl esters of NSAIDs* [Wallace JL (1994; Wallace JL (1995)]: Ibuprofen sodium salt (**1a**) (11.40 g, 0.050 mol) and 1,4-dibromobutane (43.20 g, 0.20 mol) in dry DMF (70 ml) were placed in a dry 250 ml round-bottomed flask that was equipped with a reflux condenser and a CaCl_2_ drying tube. The mixture was magnetically stirred and heated in an oil bath at 80–90 °C overnight after which the DMF was removed by distillation under reduced pressure. The residue was extracted with diethyl ether (250 ml) and washed with hydrochloric acid (2 M, 100 ml), saturated sodium hydrogen carbonate solution (50 ml) and water (100 ml), respectively. The organic layer after drying (MgSO_4_) was filtered and evaporated to yield an oily residue (23.30 g) which was shown to be impure by TLC (1:3, ethyl acetate: petroleum ether). Purification by flash column chromatography gave the bromobutyl ester of (R/S)-ibuprofen (**2a**) (*R*_f_ 0.79) (7.64 g, 45%) as a colourless oil; IR ν (tlf) 1736 cm^−1^ (> C=O); ^1^H NMR δ (CDCl_3_) 0.92 (6H, d, *J* = 7.5 Hz, 2Me), 1.50 (3H, d, *J* = 8 Hz, Me), 1.65—1.90 (5H, m, − CH_2_CH_2_- and > CH-), 2.48 (2H, d, *J* = 8 Hz, − CH_2_–Ar), 3.33 (2H, t, *J* = 7.5 Hz, − CH_2_Br), 3.70 (1H, q, *J* = 8 Hz, ArCH <), 4.15 (2H, t, *J* = 7.5 Hz, − O–CH_2_-), 7.10 (2H, d, AB system *J* = 8.5 Hz, Ar), 7.22 (2H, d, AB system *J* = 8.5 Hz, Ar); ^13^C NMR δ (CDCl_3_) 18.21, 22.24, 24.94, 27.05, 29.00, 30.04, 32.88, 44.95, 63.65, 127.35, 129.17, 137.61, 140.39, 174.53; MS m/z 340/342 (M^+^, Br^79^/Br^81^). HRMS: m/z = 340.1052 (M^+^). C_17_H_25_O_2_Br^79^ required 340.1039 (M^+^). The bromobutyl ester (**2b**) was made from (R)-(-)-ibuprofen (**1b**) (200 mg, 0.97 mmol), 60% sodium hydride dispersion in mineral oil (23.3 mg,0.97 mmol) and 1,4-dibromobutane (1.0 g, 4.6 mmol) in dry DMF (5 ml) as a colourless oil (250 mg, 75.5%), (*R*_f_ 0.64, 1:8, ethyl acetate: petroleum ether). HRMS: *m*/*z* = 340.1055 (M^+^). C_17_H_25_O_2_Br^79^ required 340.1039 (M^+^). The bromobutyl ester (**2c**) was made from (S)-( +)-ibuprofen (**1c**) (1.79 g, 8.69 mmol), 60% sodium hydride dispersion in mineral oil (350 mg,8.69 mmol) and 1,4-dibromobutane (8.0 g, 37 mmol) in dry DMF (30 ml) as a colourless oil (1.20 g, 34%). HRMS: *m*/*z* = 340.1050 (M^+^). C_17_H_25_O_2_Br^79^ required 340.1039 (M^+^).

The bromobutyl ester (**2d**) was made from naproxen (**1d**) (8.50 g, 36.9 mmol), potassium carbonate (5.50 g, 20 mmol) and 1,4-dibromobutane (32 g, 80 mmol) in dry DMF (55 ml) at 120°C overnight as a colourless oil (4.50 g, 33.5%) (*R*_f_ 0.57; 1:3, ethyl acetate: petroleum ether); ^1^H NMR δ (CDCl_3_) 1.50–1.65 (5H, m,-CH_2_ and Me), 1.80 (2H, m, − CH_2_), 3.32 (2H, t, *J* = 7.5 Hz, − CH_2_Br), 3.80–3.95 (4H, m, > CH and OMe), 4.13 (2H, t, *J* = 7.5 Hz, OCH_2_-), 7.10–7.20 (2H, m, H-5 and H-7), 7.45 (1H, d, *J* = 9 Hz, H-3), 7.65–7.75 (3H, m, H-1, H-4 and H-8); ^13^C NMR δ (CDCl_3_) 18.29, 27.07, 29.30, 32.92, 45.35, 55.16, 63.60, 105.49, 118.89, 125.80, 126.06, 127.05, 128.80, 129.14, 133.59, 135.52, 157.54, 174.47; MS *m*/*z* 364/366 (M^+^, Br^79^/Br^81^). HRMS: *m*/*z* = 364.0690 (M^+^). C_18_H_21_O_3_Br^79^ requires 364.0675 (M^+^).

The bromobutyl ester (**2e**) was made from aspirin (**1e**) (18.00 g, 0.10 mol), sodium hydride (60% dispersion in mineral oil, 4.00 g, 0.16 mol) and 1,4-dibromobutane (90.0 g, 0.41 mol) in dry DMF (100 ml) at 80 °C overnight as a colourless oil (23.0 g, 89%) (R_f_ 0.62;1:4, ethyl acetate: petroleum ether); ^1^H NMR δ (CDCl_3_) 1.70–1.80 (4H, m, − CH_2_CH_2_-), 2.30 (3H, s, Me), 3.35 (2H, t, *J* = 7.5 Hz, CH_2_Br), 4.40 (2H, t, *J* = 7.5 Hz, OCH_2_-), 7.10 (1H, d, *J* = 8.3 Hz, H-3), 7.30 (1H, t, *J* = 8.3 Hz, H-4), 7.55 (1H, t, *J* = 8.3 Hz, H-5), 7.97 (1H, d, *J* = 8.3 Hz, H-6); ^13^C NMR δ (CDCl_3_) 20.70, 27.15, 29.09, 32.87, 64.08, 123.09, 123.69, 125.87, 129.66, 131.48, 133.76, 150.55, 164.24, 169.95; MS *m*/*z* 314/316 (M^+^, Br^79^/Br^81^). HRMS: m/z = 314.0168 (M^+^).C_13_H_15_O_4_Br^79^ requires 314.0154 (M^+^).

The bromoester (**2f**) was prepared from indomethacin (**1f**) (17.86 g, 0.05 mol), potassium carbonate (7.0 g, 0.05 mol) and 1,4-dibromobutane (45.0 g, 0.20 mol) in dry DMF (70 ml) at 120 °C overnight as a cream coloured solid, m.p. 69.8–70.5 °C, (14.0 g, 57%), (*R*_f_ 0.50; 1:4, ethyl acetate: petroleum ether); ^1^H NMR δ (CDCl_3_) 1.70–1.90 (4H, m, − CH_2_CH_2_-), 2.40 (3H, s, Me), 3.36 (2H, t, *J* = 7.5 Hz, − CH_2_Br), 3.66 (2H, s, − CH_2_), 3.83 (3H, s, OMe), 4.15 (2H, t, *J* = 7.5 Hz, OCH_2_-), 6.65 (1H, d, *J* = 8.5 Hz, H–H-6), 6.87 (1H, d, *J* = 8.5 Hz, H-7), 6.97 (1H, s, H-4), 7.47 (2H, d, *J* = 8.5 Hz, ortho to Cl), 7.68 (2H, d, *J* = 8.5 Hz, ortho to N–CO); ^13^C NMR δ (CDCl_3_) 13.22, 27.14, 29.12, 30.24, 32.78, 55.59, 63.89, 101.18, 111.48, 112.39, 114.84, 128.98, 130.45, 130.67, 131.04, 133.75, 135.78, 139.11, 155.91, 168.13, 170.66; MS *m*/*z* 491.5/493.5 (M^+^, Br^79^/Br^81^). HRMS: *m*/*z* = 412.1332 (M^+^). C_23_H_23_NO_4_Cl^35^Br^79^ requires 412.1316 (M^+^).

The bromoester (**2 g**) was prepared from (R)-(-)-2-(3-fluorobiphenyl-4yl)propanoic acid (**1g**) (1.00 g, 4.09 mmol), 60% sodium hydride dispersion in mineral oil (160 mg, 4.09 mmol) and 1,4-dibromobutane (3.0 g, 13.9 mmol) in dry DMF (20 ml) at 95–100 °C overnight as a colourless oil (1.44 g) which was purified by flash column chromatography (1:9, ethyl acetate: petroleum ether) to give pure (**2 g)** (0.90 g, 58%), (R_f_ 0.60; 1:9, ethyl acetate: petroleum ether); IR ν (tlf) 1733 cm^−1^ (> C=O); ^1^H NMR δ (CDCl_3_) 1.53 (3H, d, *J* = 7.24 Hz, Me), 1.70–1.91 (4H, m, − CH_2_–CH_2_-), 3.36 (2H, t, *J* = 6.46 Hz, Br–CH_2_), 3.75 (1H, q, *J* = 7.24 Hz, > CH–CO-), 4.13 (2H, t, *J* = 6.20, O–CH_2_), 7.10–7.17 (2H, m, Ar), 7.35–7.56 (6H, m, Ar); ^13^C NMR δ (CDCl_3_) 18.58, 27.57, 29.57, 32.63, 45.37, 64.36, 115.58, 123.70, 127.86, 128.41, 128.87,129.74, 130.92, 131.10, 135.80, 142.20, 158.07, 162.00, 174.16. HRMS: *m*/*z* = 378.0647 (M^+^). C_19_H_20_O_2_FBr^79^ required 378.0632 (M^+^).

The bromoester (**2 h**) was prepared from (S)-(+)-2-(3-fluorobiphenyl-4yl)propanoic acid (**1h**) (0.50 g, 2.05 mmol), 60% sodium hydride dispersion in mineral oil (80 mg, 2.05 mmol) and 1,4-dibromobutane (1.5 g, 6.95 mmol) in dry DMF (12 ml) at 95–100 °C overnight as a colourless oil (0.80 g) which was purified by flash column chromatography (1:9, ethyl acetate: petroleum ether) to give pure **2 h** (0.52 g, 67%), HRMS: *m*/*z* = 378.0650 (M^+^). C_19_H_20_O_2_FBr^79^ required 378.0632 (M^+^).

The bromoester (**2i**) was prepared from (R/S)-2-(3-fluorobiphenyl-4yl)propanoic acid (**1i**) (4.88 g, 20 mmol), 60% sodium hydride dispersion in mineral oil (800 mg, 20 mmol) and 1,4-dibromobutane (15 g, 69.5 mmol) in dry DMF (100 ml) at 95–100 °C overnight as a colourless oil (4.20 g, 55%) which was purified from residues of 1,4-dibromobutane by evaporation under high vacuum at 100 °C and was pure according to TLC (1:9, ethyl acetate: petroleum ether).

*Typical procedure* (Wallace and Cirino 1994; Wallace et al. 1995) *for preparing the nitroxybutyl esters of the NSAIDs is illustrated by the synthesis of nitroxybutyl ester of ibuprofen* (**3a**): A mixture of ibuprofen bromobutyl ester (**2a**) (8.00 g, 0.02 mol) and silver nitrate (8.00 g, 0.04 mol) in dry distilled acetonitrile (56 ml) was stirred in an oil bath at 50 °C in a dry round bottomed flask equipped with a CaCl_2_ drying tube until TLC (1:4, ethyl acetate: petroleum ether) showed the reaction to be complete (5 h). The mixture was decanted into deionised water and extracted with DCM (150 ml). The organic layer after drying (MgSO_4_) was filtered and evaporated to yield an oily residue which was purified by flash chromatography (1:4; ethyl acetate: petroleum ether) to give nitroxybutyl ester of ibuprofen (**3a**) (R_f_ 0.68) (4.78 g, 74%) as a colourless oil, IR ν (tlf) 1733 (> C=O), 1630 cm^−1^ (ONO_2_); ^1^H NMR δ (CDCl_3_) 0.92 (6H, d, *J* = 7.5 Hz, 2Me), 1.50 (3H, d, *J* = 8 Hz, Me), 1.60–1.74 (4H, m, − CH_2_CH_2_-), 1.78–1.95 (1H, m, > CH-), 2.45 (2H, d, *J* = 8 Hz, − CH_2_-Ar), 3.70 (1H, q, *J* = 8 Hz, ArCH <), 4.05–4.18 (2H, m, − O–CH_2_-), 4.37 (2H, t, *J* = 7.5 Hz, − CH_2_ONO_2_), 7.1 (2H, d, AB system *J* = 7 Hz, Ar), 7.22 (2H, d, AB system *J* = 7 Hz, Ar); ^13^C-NMR δ (CDCl_3_) 18.16, 22.19, 24.03, 25.00, 30.02, 44.91, 63.44, 127.21, 129.17, 137.55, 140.48, 174.47; MS *m*/*z* 323 (M^+^). HRMS: *m*/*z* = 323.1740 (M^+^). C_17_H_25_NO_5_ requires 323.1734 (M^+^).

Nitroxybutyl ester (**3b**) was obtained from the bromoester (**2b**) (250 mg, 0.73 mmol) and silver nitrate (620 mg, 3.65 mmol) in acetonitrile (5 ml) after purification (1:8; ethyl acetate: petroleum ether) as a colourless oil (108 mg, 46%), (*R*_f_ 0.45). HRMS: *m*/*z* = 323.1743 (M^+^). C_17_H_25_NO_5_ requires 323.1734 (M^+^).

Nitroxybutyl ester (**3c**) was obtained from the bromoester (**2c**) (1.17 g, 3.43 mmol) and silver nitrate (3 g, 17.6 mmol) in acetonitrile (40 ml) after purification (1:8; ethyl acetate: petroleum ether) as a colourless oil (1.00 g, 90%).

Nitroxybutyl ester (**3d**) was obtained from the bromoester (**2d**) (4.47 g, 12 mmol) and silver nitrate (4.08 g, 24 mmol) in acetonitrile (40 ml) as a colourless oil (2.67 g, 64%) (R_f_ 0.55); IR ν (tlf) 1729 (> C=O), 1627 cm^−1^ (ONO_2_); ^1^H-NMR δ (CDCl_3_) 1.58 (3H, d, *J* = 7.2 Hz, Me), 1.65–1.68 (4H, m, − CH_2_CH_2_-), 3.85 (1H, q, *J* = 7.2 Hz, > CH), 3.92 (3H, s, OMe), 4.12 (2H, t, *J* = 6.3 Hz, OCH_2_-), 4.31 (2H, t, *J* = 6.3 Hz, − CH_2_ONO_2_), 7.12–7.17 (2H, m, H-7 and H-5), 7.41 (1H, d, *J* = 8.2 Hz, H-3), 7.66–7.73 (3H, m, H-1, H-4 and H-8); ^13^C-NMR δ (CDCl_3_) 18.18, 23.27, 24.73, 45.31, 55.15, 63.59, 72.19, 105.47, 118.92, 125.78, 125.97, 127.06, 128.77, 129.08, 133.59, 135.44, 157.56, 174.43; MS m/z 347 (M^+^). HRMS: *m*/*z* = 347.1375 (M^+^). C_18_H_21_NO_6_ requires 347.1370 (M^+^).

Nitroxybutyl ester (**3e**) was obtained from the bromoester (**2e**) (23.0 g, 73 mmol) and silver nitrate (24.0 g, 0.14 mol) in dry acetonitrile (180 ml) as a colourless oil (12.17 g, 56%) (R_f_ 0.45), IR ν (tlf) 1766, 1724 (> C=O), 1627 cm^−1^ (ONO_2_); ^1^H-NMR δ (CDCl_3_) 1.82–1.87 (4H, m, − CH_2_CH_2_-), 2.33 (3H, s, Me), 4.29 (2H, t, *J* = 6.3 Hz, OCH_2_-), 4.48 (2H, t, *J* = 6.3 Hz, − CH_2_ONO_2_), 7.09 (1H, dd, *J* 8.3 and 1.0 Hz, H-3), 7.30 (1H, td, *J* = 8.3 and 1.0 Hz, H-4), 7.55 (1H, td, *J* = 8.3 and 1.0 Hz, H-5), 7.98 (1H, dd, *J* = 8.3 and 1.0 Hz, H-6); ^13^C-NMR δ (CDCl_3_) 20.79, 23.41, 24.85, 30.65, 63.94, 72.47, 123.03, 123.69, 126.06, 131.36, 133.81, 150.55, 164.14, 169.42; MS *m*/*z* 297 (M^+^). HRMS: *m*/*z* = 297.0856 (M^+^). C_13_H_15_NO_7_ required 297.0849 (M^+^).

Nitroxybutyl ester (**3f**) was obtained from the bromoester (**2f**) (6.0 g, 12 mmol) and silver nitrate (4.84 g, 66 mmol) in dry acetonitrile (30 ml) as a light-brown paste (5.1 g, 89%) (R_f_ 0.55), IR ν (tlf) 1734, 1683 (> C=O), 1628 cm^−1^ (ONO_2_); ^1^H-NMR δ (CDCl_3_) 1.71–1.78 (4H, m, − CH_2_CH_2_-), 2.40 (3H, s, Me), 3.68 (2H, s – CH_2_CO), 3.84 (3H, s, OMe), 4.12–4.17 (2H, m, OCH_2_-), 4.37–4.42 (2H, m, − CH_2_ONO_2_), 6.67 (1H, dd, *J* = 9.3 and 2.6 Hz, H-6), 6.86 (1H, d, *J* = 9.3 Hz, H-7), 6.95 (1H, d, *J* = 2.6 Hz, H-4), 7.49 (2H, d of AB system, *J* = 8.7 Hz, ortho to Cl), 7.65 (2H, d of AB system, *J* = 8.7 Hz, ortho to > NCO); ^13^C-NMR δ (CDCl_3_) 13.16, 23.38, 24.80, 30.17, 55.54, 63.87, 72.37, 101.27, 111.34, 112.31, 114.83, 128.98, 130.43, 130.67, 131.02, 133.74, 135.81, 139.10, 155.90, 168.11, 170.61; MS *m*/*z* 474.5 (M^+^). HRMS: *m*/*z* = 474.1272 (M^+^). C_23_H_23_N_2_O_7_Cl^35^ required 474.11945 (M^+^).

(R)-(-)-Nitroxybutyl ester (**3 g**) was obtained from the (R)-(-)-bromoester (**2 g**) (870 mg, 2.30 mmol) and silver nitrate (3.12 g, 18.3 mmol) in dry acetonitrile (20 ml) as a colourless oil (700 mg, 84.5%), (R_f_ 0.55; 1:9, ethyl acetate: petroleum ether); [α] = − 14.87°; IR ν (tlf) 1734 (> C=O), 1627 cm^−1^ (ONO_2_); ^1^H-NMR δ (CDCl_3_) 1.51(3H, d, *J* = 7.24 Hz, Me), 1.63–1.73(4H, m, − CH_2_CH_2_-), 3.73(1H, q, *J* = 7.24 Hz, > CH-CO), 4.10(2H, t, *J* = 5.69 Hz, − OCH_2_-), 4.37(2H, t, *J* = 5.95, − CH_2_–ONO_2_), 7.07–7.17(2H, m, Ar), 7.28–7.45(4H, m, Ar), 7.48–7.55(2H, m, Ar); ^13^C-NMR δ (CDCl_3_) 18.53, 23.69, 25.20, 45.31, 64.31, 72.94, 115.53, 123.97, 127.77, 128.28, 128.55, 128.85, 129.03, 129.52, 131.11, 135.77, 142.13, 158.07, 162.00, 174.16; HRMS: *m*/*z* = 361.1364 (M^+^). C_19_H_20_NO_5_F required 361.1326 (M^+^).

(S)-( +)-Nitroxybutyl ester (**3 h**) was obtained from the (S)-( +)-bromoester (**2 h**) (500 mg, 1.30 mmol) and silver nitrate (1.76 g, 10.3 mmol) in dry acetonitrile (15 ml) after purification as a colourless oil (400 mg, 84%); [α] =  + 13.41°. HRMS: m/z = 361.1429(M^+^). C_19_H_20_NO_5_F required 361.1326 (M^+^).

(R/S)-Nitroxybutyl ester (**3i**) was obtained from the (R/S)-bromoester (**2i**) (4.20 g, 11 mmol) and silver nitrate (9.40 g, 55.3 mmol) in dry acetonitrile (100 ml) after purification as a colourless oil (3.20 g, 80%) [Wallace and Cirino 1994; Wallace et al. 1995; Menzel and Kolarz 1997).

### Pharmacology

#### Deer common digital artery contractility studies

The methods employed were those previously described (Callingham et al. 2012). The experiments were performed on isolated segments of the left common digital artery of the fallow deer (*Dama dama*) slaughtered at the Denham Park Estate in Bury St. Edmunds (UK) for venison according to E.U. Red Meat regulations. The arteries, from deer of either sex, were removed and transported over ice in vials containing physiological saline solution (PSS; composed of: NaCl 118 mM, KCl 4.57 mM, CaCl_2_ 2.5 mM, NaHCO_3_ 25 mM, MgSO_4_ 1.19 mM, KH_2_PO_4_ 1.19 mM, glucose 5.55 mM at pH 7.4, aerated with 95% O_2_ and 5% CO_2_) (Callingham et al. 2012). On arrival at the laboratory, the vessels were dissected free of extraneous tissues and stored, until required, in fresh aerated PSS at 4 °C. With changes of PSS daily, the vessels remained viable for up to 10 days.

Segments (approximately 3 mm in length), were mounted, in 10 ml, water-jacketed organ baths at 37 °C and attached to Harvard isometric transducers (0–50 g sensitivity), connected, via Harvard amplifiers and A/D converters (PowerLab® 8/35, ADInstruments Ltd, Bishops Mews, Transport Way, Oxford, OX4 6HD, UK) for computer recording of developed tension. Resting tension was adjusted to 3 g, which was maintained during a 45 min period of acclimatisation and beyond. The integrity of the vascular endothelium was tested by measuring the relaxation produced by addition of 10^–6^ M histamine to segments pre-contracted with either 10^–6^ M 5-HT or 10^–6^ M PHE; since acetylcholine, the agent normally employed to detect functional endothelium is without effect in this preparation. In each experiment, the vessel rings were contracted, either with single concentrations or graded concentrations of (5-HT).

Cumulative changes in tension to applied agents were plotted as percentages of maximum responses against log concentrations of the relevant agent and fitted to the Hill equation by use of the non-linear regression facility in Kaleidagraph® (Synergy Software, 2457 Perkiomen Ave., Reading PA, USA 19,606) with n-values referring to the number of animals used. Tests for statistical significance were performed using the unpaired t-test.

In Figs. 3, 4, 5, 6, 7 inclusive, parameters for 5-HT (EC_50_ ± s.e.m. and maximum tension ± s.e.m.) were obtained from the mean regressions. In Figs. 8, 9, 10, 11 inclusive, while the regressions were derived as above, tests for statistical significance were applied to individual mean data points and identified by asterisks as appropriate.

#### Drugs and reagents

Stock solutions of the NSAIDs were made by first dissolving a few milligrams of the compound in 0.25 ml of DMSO (dimethyl sulphoxide) and made up to 10^–2^ M with an appropriate volume of deionised water. These solutions, together with any dilutions, were kept on ice until used.

This investigation tested four NSAIDs (aspirin, ibuprofen, naproxen, and indomethacin) and four corresponding NO-donating NSAIDs (aspirin nitroxybutyl ester, ibuprofen nitroxybutyl ester, naproxen nitroxybutyl ester and indomethacin nitroxybutyl ester).

Stock solutions of 10^−2 ^M 5-HT), phenylephrine (PHE) and histamine were prepared and kept at 4 °C and diluted with deionised water for use on the day of the experiment and kept on ice. Solutions of methylene blue for use as an inhibitor of nitric oxide synthase (Mayer et al. 1993) were made up on the day they were required.

#### Experimental protocol

Rings of 2–3 mm length were cut from the digital arteries using scissors and mounted in the organ bath by sliding the two hooks into the lumen of the artery. Each water bath was filled with PSS (buffered salt solution) and continuously aerated with 95% O_2_ 5% CO_2_. The jackets surrounding the water baths had water heated to 37 °C continuously pumped through them to maintain physiological temperature in the water baths. The day’s stock solution flask of aerated PSS was also kept submerged in the water bath so that it was at the correct temperature when it was added to the organ baths. The tension pulled by the rings was adjusted to 3 g before each experiment was begun.

On the morning of each day of experiments, the artery segments were pre-contracted with 10^−5^ M 5-HT as this concentration was sufficient to achieve the maximum contractile response; previous studies had shown to induce the rings to respond well to subsequent drug additions. When the vessels had reached maximum contraction, 10^−6^ M histamine was added to the organ baths to test for the presence of a functional endothelium. The organ baths were then washed out and filled with fresh PSS. The rings were left to relax for an hour, with the tension returned to 3 g at intervals and the experiment proper was begun.

#### Data recording

The transducers were calibrated by use of the PowerLab® calibration facility and tested for linearity of response by attaching weights from 1 to 20 g. All data were processed by use of LabChart® (ADInstruments) on the recording computer.

The cumulative changes in tension to applied agents were plotted as percentages of maximum responses against log concentrations of the relevant agent and fitted to the Hill equation by non-linear regression, with n-values referring to the number of animals used. Only rings from left feet were used after ensuring, having previously that there were no differences in responses between rings taken from either foot, to ensure that the n-values truly represented individual animals. Tests for statistical significance were performed using the unpaired t-test.

## Results

**Fig. 3:**
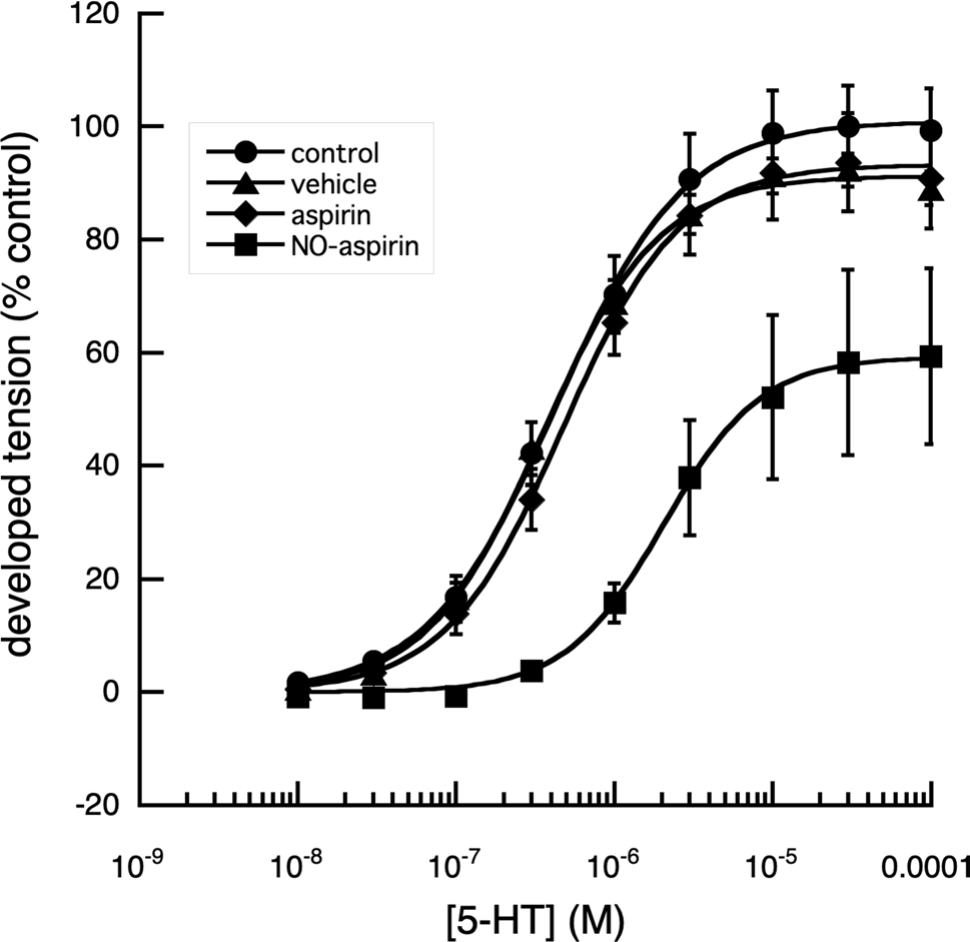
Effect of aspirin and NO-aspirin on the cumulative log[concentration]—vasoconstrictor responses of fallow deer isolated arterial rings to 5-HT. A comparison of the contractile responses of arterial rings to 5-HT in the presence and absence of aspirin (10^–4^ M) and NO-aspirin (10^–4^ M), showed that aspirin had no significant effect on the responses of the arterial rings to 5-HT (*P* > 0.05), while NO-aspirin, significantly reduced the maximum tension produced (*P* < 0.001) together with a significant increase in the EC_50_ of applied 5-HT (*P* < 0.001), when compared with responses of control rings and rings in the presence of aspirin. Control: *n* = 19, EC_50_ = 4.29 × 10^–7^ ± 1.63 × 10^–8^ M, max. developed tension (percent) = 100.9 ± 0.81. Vehicle: *n* = 8, EC_50_ = 3.53 × 10^–7^ ± 2.61 × 10^–8^ M, max. developed tension (percent) = 91.24 ± 1.12. Aspirin: *n* = 8, EC_50_ = 4.74 × 10^–7^ ± 1.88 × 10^–8^ M, max. developed tension (percent) = 93.43 ± 1.40. NO-Aspirin: *n* = 12, EC_50_ = 2.05 × 10^–6^ ± 1.02 × 10^–7^ M, max. developed tension (percent) = 59.19 ± 0.83. There was no significant difference between the responses of control vessels and those to which vehicle had been added volumes appropriate to the concentrations of applied drugs (*P* > 0.05)

**Fig. 4:**
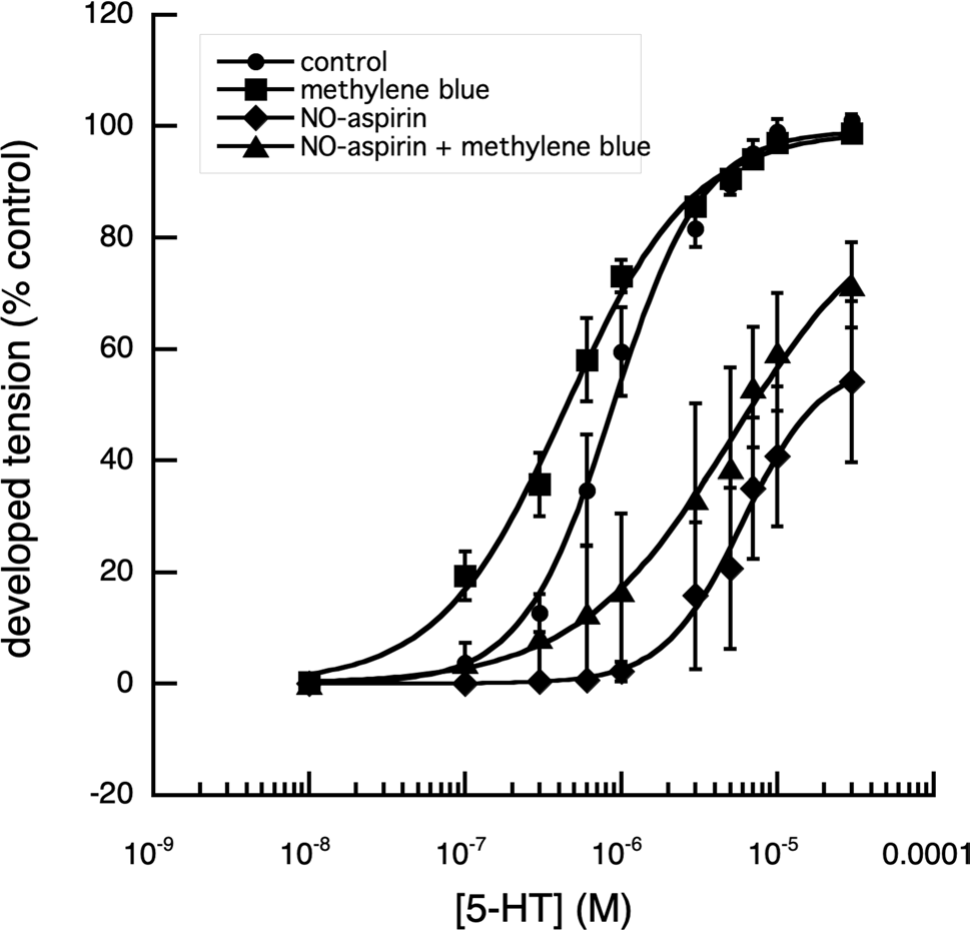
Effect of methylene blue on the cumulative log[concentration]—vasoconstrictor responses of fallow deer isolated arterial rings to 5-HT. In the presence of 10^–4^ M methylene blue, a direct nitric oxide synthase and guanylyl cyclase inhibitor (Mayer et al. 1993), the contractile responses of the arterial rings to 5-HT were enhanced, with a significant decrease (*P* < 0.001) of the EC_50_ value, without effect on the maximum tension developed. In the absence of methylene blue, NO-aspirin produced a significant reduction in the maximum response to 5-HT (*P* < 0.01) and a significant increase in EC_50_ value of 5-HT (*P* < 0.001). The presence of methylene blue, had no significant effect on the NO-aspirin induced increased EC_50_ value but appeared to reduce its reduction of the maximum effect of 5-HT. Control: *n* = 3, EC_50_ = 8.83 × 10^–7^ ± 6.83 × 10^–8^ M, max. developed tension (percent) = 99.26 ± 2.43. Meth Blue: *n* = 3, EC_50_ = 4.44 × 10^–7^ ± 3.16 × 10^–8^ M, max. developed tension (percent) = 99.19 ± 1.90. NO-Aspirin: *n* = 3, EC_50_ = 5.99 × 10^–6^ ± 5.15 × 10^–7^ M, max. developed tension (percent) = 57.69 ± 3.03. Meth Blue & NO-aspirin: *n* = 3, EC_50_ = 5.25 × 10^–6^ ± 1.36 × 10^−6^ M, max. developed tension (percent) = 88.97 ± 8.28

Nitroxybutyl-aspirin (NO-aspirin) effectively reduced the contractile responses of digital artery segments produced by increasing concentrations of 5-HT, whereas aspirin was without effect (Fig. 3); as was aspirin butyl ester (data not shown). When used at a concentration of 10^–4^ M, NO-aspirin increased the EC_50_ of 5-HT to 9.1 × 10^–7^ ± 0.7 × 10^–8^ M (*n* = 3) from 5.2 × 10^–7^ ± 0.7 × 10^–8^ M. (*n* = 3). However, when this experiment was repeated to ascertain if 10^–4^ M methylene blue would reduce the effectiveness of NO-aspirin by sequestering the released NO, there was no significant change in EC_50_ values, which were control; 8.8 × 10^–8^ ± 0.7 × 10^–8^ M, methylene blue alone; 4.4 × 10^–7^ ± 0.3 × 10^–8^ M (n = 3), NO-aspirin alone; 5.99 × 10^–6^ ± 0.52 × 10^–6^ M (*n* = 3) and NO-aspirin plus methylene blue; 5.25 × 10^–6^ ± 1.36 × 10^–6^ M (*n* = 3), (Fig. 4).

**Fig. 5:**
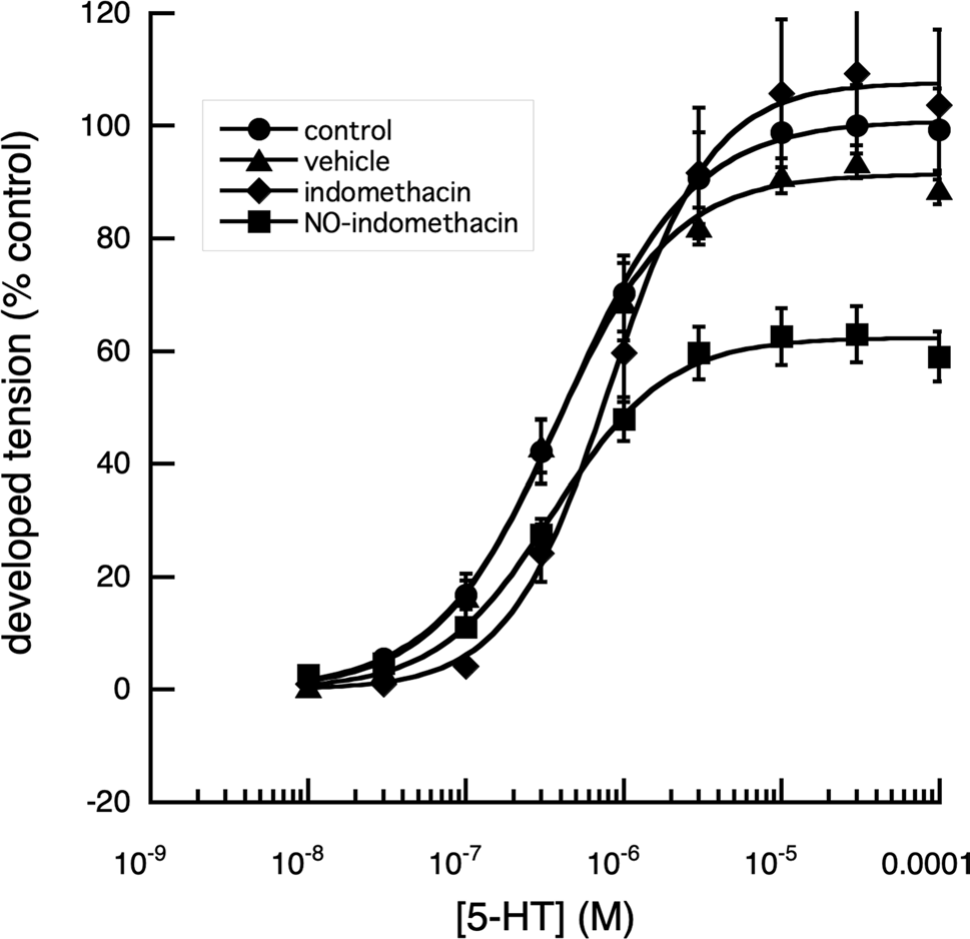
Effect of indomethacin and NO-indomethacin on the cumulative log[concentration]—vasoconstrictor responses of fallow deer isolated arterial rings to 5-HT. A comparison of the contractile responses of arterial rings to 5-HT in the presence and absence of indomethacin (10^–4^ M) and NO-indomethacin (10^–4^ M,) showed that while indomethacin had no significant effect on the responses of the arterial rings to 5-HT (*P* > 0.05), NO-indomethacin, significantly reduced the maximum tension produced (*P* < 0.001), but without significant effect on the EC_50_ value when compared with responses of control rings and rings in the presence of indomethacin. Control: *n* = 19, EC_50_ = 4.29 × 10^–7^ ± 1.63 × 10^–8^ M, max. developed tension (percent) = 100.9 ± 0.81. Vehicle: *n* = 8, EC_50_ = 3.53 × 10^–7^ ± 2.61 × 10^–8^ M, max. developed tension (percent) = 91.43 ± 1.40. Indomethacin: n = 8, EC_50_ = 8.20 × 10^–7^ ± 4.78 × 10^–8^ M, max. developed tension (percent) = 107.6 ± 1.55. NO-Indomethacin: *n* = 9, EC_50_ = 3.56 × 10^–7^ ± 3.17 × 10^–8^ M, max. developed tension (percent) = 62.37 ± 1.81

**Fig. 6:**
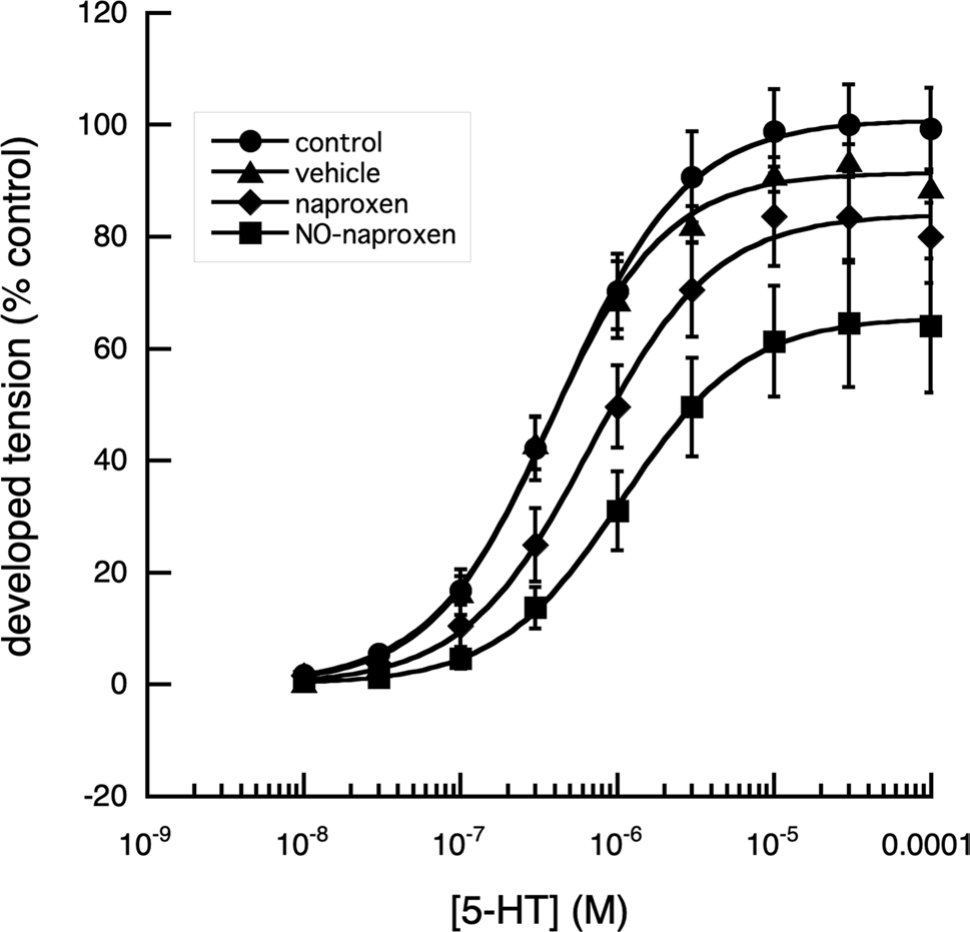
Effect of naproxen and NO-naproxen on the cumulative log[concentration]—vasoconstrictor responses of fallow deer isolated arterial rings to 5-HT. A comparison of the contractile responses of arterial rings to 5-HT in the presence and absence of naproxen (10^–4^ M) and NO-naproxen (10^–4^ M), showed that naproxen had no significant effect on the responses of the arterial rings to 5-HT, while NO- naproxen, significantly reduced the maximum tension produced (*P* < 0.05) together with a significant increase in the EC_50_ of applied 5-HT (*P* < 0.001), when compared with responses of control rings. However, there were no significant differences between NO-naproxen and naproxen (*P* > 0.05). Control: *n* = 19, EC_50_ = 4.29 × 10^–7^ ± 1.63 × 10^–8^ M, max. developed tension (percent) = 100.9 ± 0.81. Vehicle: *n* = 8, EC_50_ = 3.53 × 10^–7^ ± 2.61 × 10^–8^ M, max. developed tension (percent) = 91.43 ± 1.40. Naproxen: *n* = 8, EC_50_ = 6.66 × 10^–7^ ± 5.99 × 10^–8^ M, max. developed tension (percent) = 84.0 ± 1.68. NO-Naproxen: *n* = 12, EC_50_ = 1.07 × 10^–6^ ± 3.84 × 10^−8^ M, max. developed tension (percent) = 65.63 ± 0.56

**Fig. 7:**
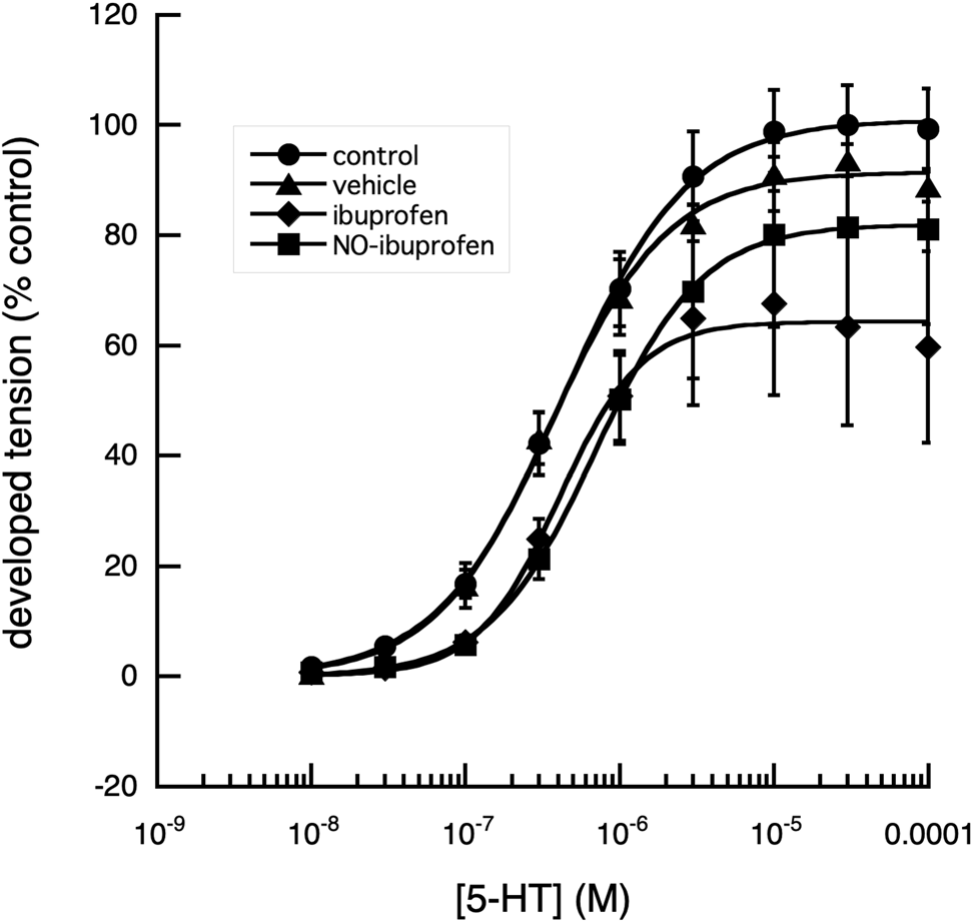
Effect of ibuprofen and NO-ibuprofen on the cumulative log[concentration]—vasoconstrictor responses of fallow deer isolated arterial rings to 5-HT. A comparison of the contractile responses of arterial rings to 5-HT in the presence and absence of ibuprofen and NO-ibuprofen show no significant difference between the effects of the two drugs on the rings’ responses to 5-HT (*P* > 0.05). At 5-HT concentrations of 10^−6^ M and 10^−4^ M the contraction in the presence of NO-ibuprofen is not significantly different from that of the control. Interestingly the results for classic ibuprofen show that the maximum contraction reached in the presence of this drug is less than that in the presence of NO-ibuprofen. Cumulative log[concentration]-response curve of the deer digital artery to 5-HT in the presence and absence of ibuprofen and ibuprofen nitroxybutyl ester. Control: *n* = 19, EC_50_ = 4.29 × 10^–7^ ± 1.63 × 10^–8^ M, max. developed tension (percent) = 100.9 ± 0.81. Vehicle: *n* = 8, EC_50_ = 3.53 × 10^–7^ ± 2.61 × 10^–8^ M, max. developed tension (percent) = 91.43 ± 14.0. Ibuprofen: *n* = 7, EC_50_ = 4 .04 × 10^–7^ ± 4.19 × 10^−8^ M, max. developed tension (percent) = 87 ± 0.21. (NO-Ibuprofen: *n* = 12, EC_50_ = 7.02 × 10^–7^ ± 1.78 × 10^−8^ M, max. developed tension (percent) = 81.9 ± 0.48

When the maximum tension that could be developed by the segments, in response to applied 5-HT, was examined, the relaxation in tension produced by NO-aspirin alone was reduced from 50 to 30% in the presence of methylene blue (Fig. 4). Of the other NSAIDs and their nitroxy-derivatives, examined, indomethacin and naproxen, produced similar results (Figs. 5, 6, 7).

However, when the effects produced by *racemic (rac)-*ibuprofen and nitroxybutyl-ibuprofen (NO-ibuprofen) were compared, on 5-HT pre-contracted arterial segments, both were effective at reducing the responses to electrical stimulation, with no significant difference (*p* > 0.05) in effect between them (Fig. 7). Another phenyl-propioinic acid, *rac-*flurbiprofen and its nitroxybutyl derivative (NO-flurbiprofen) produced similar results (data not shown). It was also found that *rac*-ibuprofen produced a reversible relaxation of vessel segments, when they had been pre-contracted with 3 × 10^–6^ M phenylephrine (PHE), to a maximum tension of 16.5 ± 15% of control, with an EC50 of 2.97 × 10^–4^ ± 10^–5^ M (*n* = 7: *p* < 0.01). In view of this unexpected relaxation produced by ibuprofen and flurbiprofen, further experiments were done to attempt to discover their mode of action.

**Fig. 8:**
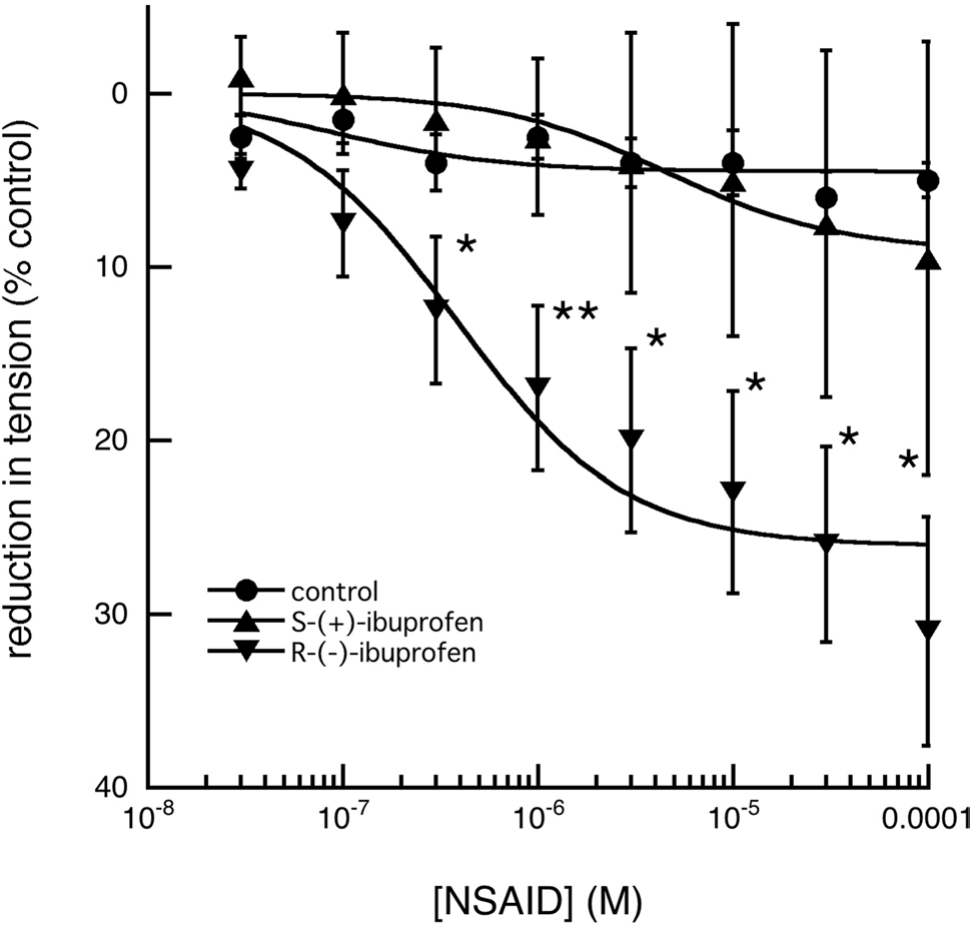
Effect of increasing concentrations of S-( +)-ibuprofen and R-(-)-ibuprofen on the tension produced in fallow deer isolated arterial rings by a constant concentration of 3 × 10^–6^ M 5-HT. S-( +)-ibuprofen, in concentrations up to 10^–4^ M had no significant effect on the maintained tension (*n* = 4; *P* > 0.05), but R-(−)-ibuprofen had a significant relaxant effect, first seen at 5 × 10^–7^ M (*n* = 6; *P* < 0.05). (control tension: *n* = 6 and points of significance are shown as: *P < 0.05, **P < 0.01)

**Fig. 9:**
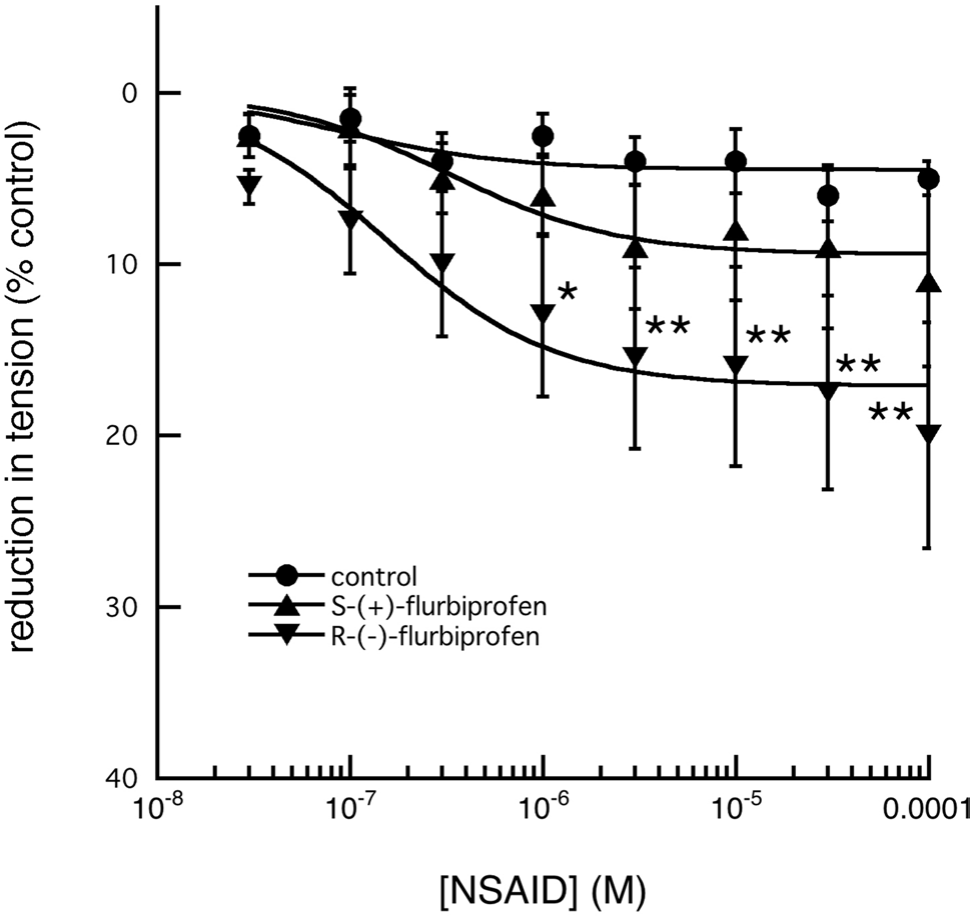
Effect of increasing concentrations of S-( +)-flurbiprofen and R-(−)-flurbiprofen on the tension produced in fallow deer isolated arterial rings by a constant concentration of 3 × 10^–6^ M 5-HT. S-( +)-flurbiprofen, in concentrations up to 10^–4^ M had no significant effect on the maintained tension (*n* = 6; *P* > 0.05), but R-(−)-flurbiprofen had a significant relaxant effect, first seen at 10^–6^ M (*n* = 8; *P* < 0.05). (control tension: *n* = 6 and points of significance are shown as: *P < 0.05, **P < 0.01)

Furthermore, since ibuprofen and flurbiprofen are diastereo-isomeric *(racemic)*-mixtures, it was decided to examine the relaxant effects of their individual enantiomers on 5-HT pre-contacted arterial segments. In both cases, the R-(−)-enantiomers were significantly (*p* < 0.01) more potent than the corresponding S-( +)-isomers (Figs. 8, 9).

**Fig. 10:**
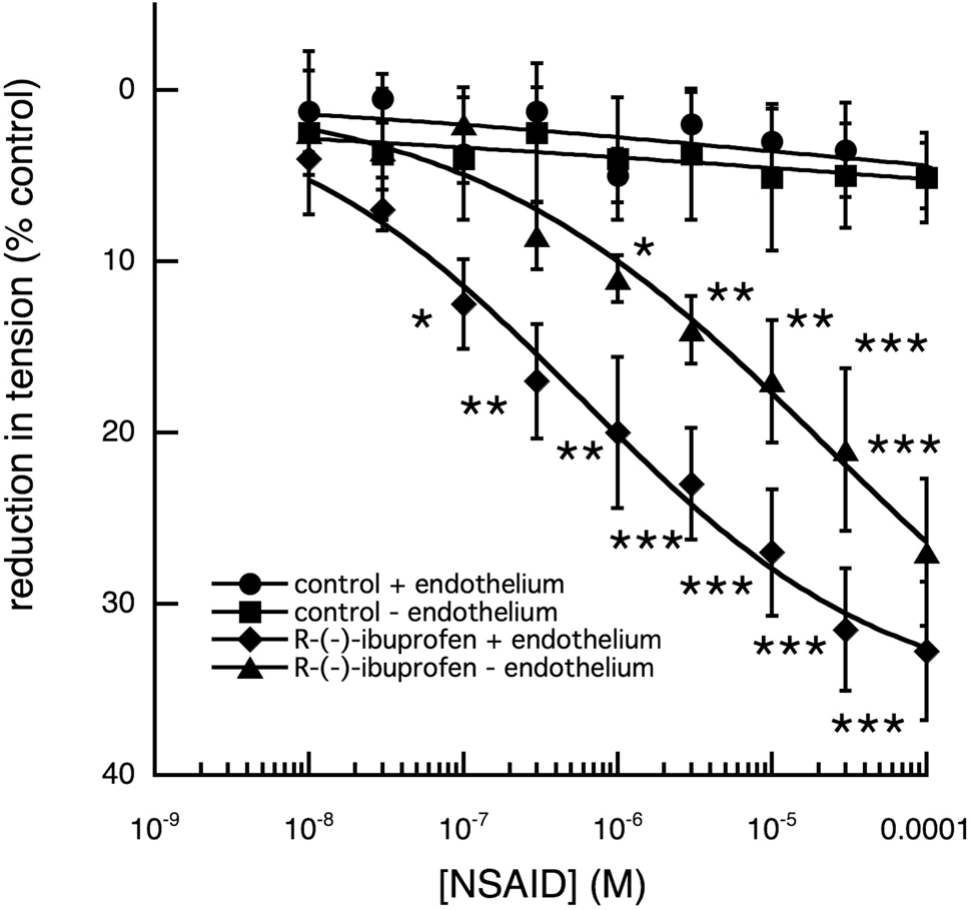
Effect of increasing concentrations of R-(−)-ibuprofen on the tension produced in fallow deer isolated arterial rings by a constant concentration of 3 × 10^–6^ M 5-HT, in the presence and absence of vascular endothelium. While there appears to be no significant difference (*P* > 0.05) between the maximum relaxation produced by R-(−)-ibuprofen in the presence (*n* = 6) and absence (*n* = 5) of endothelium, at lower concentrations the difference is significant. At 10^–7^ M R-(−)-ibuprofen the relaxation in the presence of endothelium is significant (*P < 0.05), while in its absence it is not; a difference even more marked at 3 × 10^–7^ M. (control tension: *n* = 6, the asterisks denote levels of significance between drug treated and control)

Removal of the vascular endothelium (a source of NO) reduced (*p* < 0.001) but did not eliminate the vasodilator actions of R-(−)- ibuprofen (Fig. 10.), suggesting a role for NO in the relaxation produced.

**Fig. 11:**
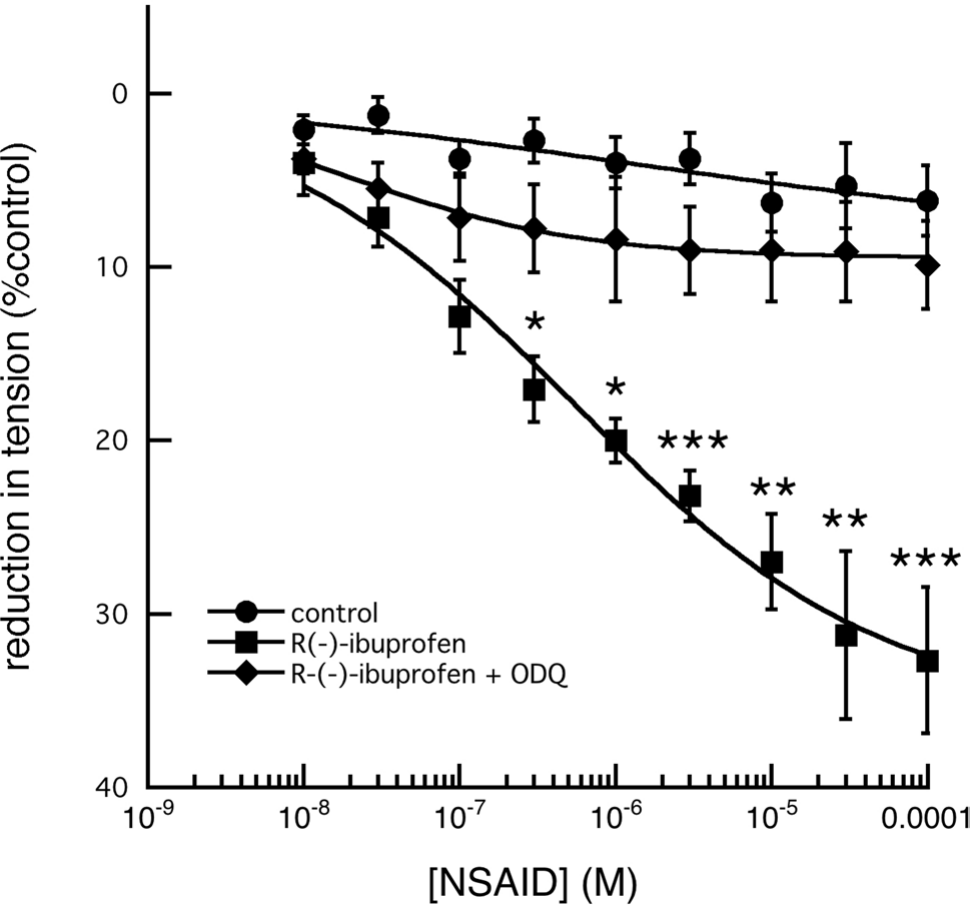
Effect of 1H-[1,2,4]oxadiazolo[4,3-a]quinoxalin-1-one (ODQ) on the relaxation of tension by increasing concentrations of R-(−)-ibuprofen on the tension produced in fallow deer isolated arterial rings by a constant concentration of 3 × 10^–6^ M 5-HT. In contrast to the highly significant relaxation of tension produced by R-(−)-ibuprofen alone of the maintained control tension (*P* < 0.01, by comparison of the regressions, R-(−)-ibuprofen: *n* = 6 and control *n* = 6), the presence of 10^–6^ M ODQ (*n* = 6) caused complete inhibition to control levels. (asterisks denote: *P < 0.05, **P < 0.01, ***, 0.001)

These relaxant effects were reduced to near control values by the soluble guanylate cyclase (sGC) inhibitor, 1H-[1,2,4]-oxadiazolo[4,3-a]quinoxalin-1-one (Feelisch et al. 1999; ODQ: 1 × 10^–5^ M) (Fig. 11).

## Discussion

These results demonstrate that the NO-donating analogues of aspirin, indomethacin, etc., significantly reduced the contractile responses of vascular smooth muscle to electrical stimulation and to applied 5-HT and PHE (results not shown), while, with the exception of ibuprofen and flurbiprofen, the parent NSAIDs were without effect. It was also shown that methylene blue (an inhibitor of NO action) significantly reduced the effect of NO-aspirin (Fig. 4), as well as other NO-NSAIDs (data not shown). In addition, the presence of haemoglobin had the same effect on NO-aspirin. This suggests that, in the presence of blood, in particular, the actions of NO-NSAIDs could be limited (data not shown).

The fact that R-(−)-ibuprofen produced a relaxation of a similar magnitude to racemic NO-ibuprofen suggests that either R-(−)-ibuprofen released NO on a similar scale to NO-ibuprofen, or that it caused relaxation by some other means. There are several other means possible, including the induction of iNOS or direct activation of soluble guanylate cyclase. Some previous work has been done on the possible involvement of ibuprofen with iNOS. One study suggests that the concentration of NO in cells can be raised by the presence of ibuprofen, through the induction of iNOS (Menzel and Kolarz 1997). This showed that, at therapeutically attainable concentrations (1–30 μM), iNOS was induced similarly by both stereoisomers of ibuprofen, although only slightly more by the R-(-)-enantiomer. In another study, ibuprofen significantly increased the spontaneous production of NO, which was unaffected by an iNOS inhibitor, suggesting instead that eNOS was involved (Miyamoto et al. 2007). This is relevant to the present study due to the observations that while S-( +)-ibuprofen was shown to have relatively little effect, this was not significantly different from the vehicle control and R-(−)-ibuprofen caused appreciable relaxation. However, contrary to this, there is evidence to suggest that ibuprofen, in fact, reduces NO produced in stressful situations, for example in the presence of bacterial endotoxin, where increased NO production leads to a fall in mean arterial blood pressure. Ibuprofen blunts this effect, and the data suggests that ibuprofen down-regulates NO production in human subjects (Vandivier et al. 1999).

The reduction in the relaxation caused by *rac*-ibuprofen was blocked by ODQ (Feelisch et al. 1999) (Fig. 11), strongly suggests that the relaxation is mediated through cGMP. Removing the endothelium of the vessels, which should prevent any action of NOS, had no significant effect on the relaxation. Attempts to employ L-nitro arginine (L-NAME) to block endogenous NO production have been complicated by its action (after potentiating contraction as expected due to the reduction in local NO) to cause a reduction in tension on its own.

By comparison, another diastereoisomeric propionic acid, *rac*-flurbiprofen had similar properties to the ibuprofen isomers, with the R-(–) enantiomer causing significantly greater relaxation than the S-( +)- enantiomer; the magnitude of the relaxation produced being less than with the same concentrations of ibuprofen enantiomers. The other difference is that the NO-flurbiprofen compounds appear to have a more potent vasorelaxant effect than the parent compound. This might be due to an increased ability to release NO. There is little difference between the magnitude of reduction in response by the two enantiomers of the NO-flurbiprofen, suggesting that they can release NO while not directly activating sGC. If activating sGC were important in their action, it would be expected that the R-enantiomer would have a greater effect than the S-enantiomer. As this is not the case, it seems likely that they are producing relaxation via NO.

The results overall suggest that R-(–)-ibuprofen directly activates sGC. They also suggest that NO-ibuprofen does not work in the same fashion. If it did then it would be likely to produce greater relaxation given its coupling with a nitric oxide-releasing moiety. The combination of the release of NO and direct activation of sGC by ibuprofen should produce a greater relaxation than just the activation of sGC alone but it does not, suggesting that the change in the chemical composition by esterification causes sufficient change in structure to prevent the compound working in the same way as R-(−)-ibuprofen. As S-( +)-ibuprofen is much less effective than the R(−)-isomer, the activation must be very specific. Due to the similarity between flurbiprofen and ibuprofen, it is no surprise that the former causes relaxation. There is also the possibility that other heme proteins are involved. It has been suggested that ODQ is non-selective and may inhibit enzymes other than sGC (Feelisch et al. 1999). This implies that NO-generating enzymes could be activated by R-(−)-ibuprofen, the effect of which is then blocked by ODQ. However, most sources claim that ODQ is a specific sGC inhibitor. An assay directly testing the effect of R-(−)-ibuprofen and flurbiprofen on the activity of guanylate cyclase could verify this claim. In a clinical setting, this discovery could prove useful if the concentrations required sufficiently to activate sGC are within a normal therapeutic range. If so, an ibuprofen preparation made up with a larger percentage of R-(−) could cause vasodilatation allowing clearance of the drug from the stomach, possibly preventing damage. After this, the drug would be converted to the active, COX inhibiting S-( +)-enantiomer, having already had the desired gastroprotective effect. A proportion of S-( +)-ibuprofen would also be available for immediate anti-inflammatory effect without waiting for conversion to take place. However, topical formulations of R-(−)-ibuprofen might have significant advantages compared with those of diclofenac, but without the excessive gastro-toxicity of the latter (Rainsford 2009, 2012).
